# Supplementary material for: Development of chemokine network inhibitors using combinatorial saturation mutagenesis
Source: Commun Biol. 2025 Apr 3;8:549. doi: 10.1038/s42003-025-07778-6 (PMC11969024; doi:10.1038/s42003-025-07778-6)
Supplement: Supplementary file 4 — Supplementary Data 2 [file 42003_2025_7778_MOESM4_ESM.zip › SupplementaryData3/scripts/xattune_ci.tar!/./script/analysis/JK/R20_CCL19_HD2X_plot.pdf]

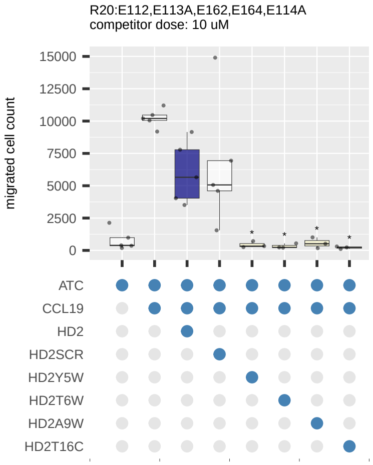
R20:E112,E113A,E162,E164,E114A
competitor dose: 10 uM
migrated cell count
15000
12500
10000
7500
5000
2500
0
*
*
*
*
ATC
CCL19
HD2
HD2SCR
HD2Y5W
HD2T6W
HD2A9W
HD2T16C
